# Wave Attenuation in Additively Manufactured Polymer Acoustic Black Hole Structures Considering the Viscoelastic Effect

**DOI:** 10.3390/polym15112457

**Published:** 2023-05-25

**Authors:** Wei Huang, Hongli Ji, Ye Ding, Jinhao Qiu

**Affiliations:** 1 School of Mechanical Engineering, Nanjing University of Science and Technology, Xiaolingwei 200, Nanjing 210094, China; huangwei91@njust.edu.cn; 2 State Key Laboratory of Mechanics and Control of Mechanical Structures, Nanjing University of Aeronautics and Astronautics, Yudao Street 29, Nanjing 210016, China; 3 State Key Laboratory of Mechanical System and Vibration, Shanghai Jiao Tong University, Dongchuan Road 800, Shanghai 200240, China

**Keywords:** acoustic black hole, polymer structures, broadband wave attenuation, viscoelastic effect, additive manufacturing

## Abstract

The acoustic black hole (ABH) is a feature commonly found in thin-walled structures that is characterized by a diminishing thickness and damping layer with an efficient wave energy dissipation effect, which has been extensively studied. The additive manufacture of polymer ABH structures has shown promise as a low-cost method to manufacture ABHs with complex geometries, exhibiting even more effective dissipation. However, the commonly used elastic model with viscous damping for both the damping layer and polymer ignores the viscoelastic changes that occur due to variations in frequency. To address this, we used Prony exponential series expansion to describe the viscoelastic behavior of the material, where the modulus is represented by a summation of decaying exponential functions. The parameters of the Prony model were obtained through experimental dynamic mechanical analysis and applied to finite element models to simulate wave attenuation characteristics in polymer ABH structures. The numerical results were validated by experiments, where the out-of-plane displacement response under a tone burst excitation was measured by a scanning laser doppler vibrometer system. The experimental results illustrated good consistency with the simulations, demonstrating the effectiveness of the Prony series model in predicting wave attenuation in polymer ABH structures. Finally, the effect of loading frequency on wave attenuation was studied. The findings of this study have implications for the design of ABH structures with improved wave attenuation characteristics.

## 1. Introduction

The efficient wave energy attenuation effect achieved in acoustic black hole (ABH) structures has been extensively studied over the last two decades [1,2,3]. It is commonly implemented by coating a small amount of viscous damping material on a host thin-walled metal structure embedded with a specific wedge or pit area, where the local inhomogeneous area with gradually diminishing thickness provides high energy density [4,5,6,7]. The concept of ABH has been attempted in industrial structures, such as turbine blades with tapered ABH edges, which exhibit effective damping within the desired frequency range [8]. Since then, researchers have explored various designs of ABH structures, including those with thin edges [9], cut-outs [10], or locally thin areas [11,12,13,14], for different applications. Parametric studies [15,16] have been conducted and optimization methods [7,17,18] developed to guide the design of ABH structures. These studies have highlighted the significant impact of geometry and the distribution of damping materials on the ABH effect. The broadband lightweight damping effect in ABH structures has shown promising potential in various applications, particularly in vibration and noise suppression.

In order to ensure high efficiency of the energy attenuation effect in ABHs, the minimum or truncation thickness must be extremely thin [19,20], which can be challenging to achieve using traditional machining methods. However, recent advancements in additive manufacturing (AM) provide useful and economic methods for creating prototypes of structures with complex geometries [21,22,23]. Furthermore, recent research on 3D-printed polymer ABH structures has shown more effective energy dissipation [24,25]. It is worth noting that both the damping material in conventional ABH structures and the polymer material of printed ABH structures are typical viscoelastic materials. The viscoelastic effects in materials with high energy loss properties are even more important, particularly in applications where frequencies or temperatures change significantly. The effective utilization of viscous materials for ABH structures requires a thorough understanding of wave propagation and attenuation, considering the viscoelastic effect.

To model the dissipation of the viscous material in ABH structures, there are several common models utilized in the literature. One such model is the complex modulus model, which is used to represent the phase lag that produces a hysteresis effect resulting in energy dissipation [26]. This model uses a complex number to describe the relationship between stress and strain, which is independent of the frequency variation. Another common model is Rayleigh damping, which is proportional to a linear combination of mass and stiffness and is generally applied in the time or frequency domain for analyzing the dynamics of ABH structures with viscous materials. Although it varies with frequency, this type of damping is not directly related to any physical process. Other damping models are used to account for frequency variation, such as the GHM method [23,27,28], the fractional derivative model [29], the anelastic displacement fields model [30], and the Prony series model [31,32]. These methods are able to model the frequency-dependent loss factor inherent in viscoelastic materials. To produce mass, stiffness, and damping matrices that can be integrated into existing finite element (FE) theory or analytical models, the necessary information required is fitting the complex modulus data to the form of material property expressions used in those methods.

A proper material model and accurate determination of its parameters are essential for achieving reliable and precise dynamic analysis of viscoelastic materials. Prony series-based viscoelastic models are widely used due to their ease of implementation in finite element analysis codes. Chen presented a method for determining the Prony series parameters of viscoelastic material using least-squares fitting to a hereditary integral model based on load versus time test data for different rate loading segments [33]. Shil’ko developed a method for determining rheological parameters of the Prony model describing the viscoelastic deformation process based on dynamic mechanical analysis, which was verified by static and dynamic mechanical tests of polymer composites [34]. Endo identified the Prony series parameters by fitting numerical and experimental creep test data of fiber-reinforced thermoplastic material [35]. Johannesmann determined the elastic and viscoelastic material parameters of a polymeric sample using broadband acoustic plate waves in an inverse procedure [36]. Barrientos and Cui presented an optimization method for obtaining viscoelastic constitutive parameters, which was successfully verified in both time-domain and frequency-domain experimental data [37,38]. However, the viscoelastic effect of materials in ABH structures has not been adequately studied thus far.

This paper focuses on studying wave attenuation in polymer ABH beams manufactured by 3D printing and accounting for the viscoelastic effect. The paper is organized as follows: In Section 2, the characteristics of the printed material are investigated. The Prony series model is employed to describe the viscoelastic behavior of the polymer. Parameters of the Prony series model are determined using an optimization method based on an experimental measurement of the material’s storage modulus and loss modulus via dynamic mechanical analysis (DMA). In Section 3, material characterization is experimentally verified using a scanning laser doppler vibrometer (SLDV) system. This provides reliable parameters for FE simulation. Section 4 investigates wave attenuation in printed ABH structures considering the viscoelastic effect, both numerically and experimentally. Reflection coefficients in the ABH beam are obtained to evaluate the wave attenuation effect. Finally, Section 5 presents the conclusions.

## 2. Materials and Methods

Viscoelastic materials, such as 3D-printed polymers, exhibit unique behaviors that combine the characteristics of both an elastic solid and a viscous fluid. These materials have time-dependent mechanical properties, including their modulus. When a viscoelastic material is subjected to harmonic excitation, it undergoes a phase lag in the strain. This feature makes viscoelastic materials ideal for energy dissipation applications. In this study, the wave attenuation effect in AM polymer ABH structures was investigated by employing the Prony series model to represent the viscoelastic effect. In this section, the viscoelastic constitutive parameters were determined, which is necessary for studying wave attenuation in printed ABH structures using numerical simulations.

### 2.1. Prony Series Model of Viscoelastic Material

For a linear vibrating system with viscoelastic damping material, assuming that the loading starts at zero time and the strain is continuous, its motion is governed by the following equation:

(1) $$\sigma \left(t\right)=G\left(t\right)\varepsilon \left(0\right)+\int_{0}^{t}G\left(t-\tau \right)\frac{d}{d\tau }\varepsilon \left(\tau \right)d\tau \;,$$

where σ is the applied stress, ε is the resulting strain, and $G\left(t\right)$ is the material dynamic modulus. In a relaxation test, the elastic and viscous mechanisms are responsible for the initial stress and the stress relaxation in the hold phase, respectively. During the stress relaxation stage, the stress needed for maintaining the constant strain decreases as the time passes by. That is to say, the modulus varies over time. There is a number of models to describe the time dependent properties. A widely used approach is called the Prony exponential series expansion, where the dynamic modulus $G\left(t\right)$ could be represented by a summation of decaying exponential functions, i.e.,

(2) $$G\left(t\right)=G_{0}-\sum_{i=1}^{N}G_{i}\left[1-e^{-t/\tau_{i}}\right]\;,$$

where $G_{0}$ is the instantaneous modulus, and $G_{i}$ and $\tau_{i}$ are the Prony coefficients and the relaxation times, respectively.

Appling a harmonic load with and oscillation frequency ω and an amplitude ${\bar{\varepsilon }}_{0}$, the stress and strain satisfy the following expression:

(3) $$\tilde{\sigma }\left(t\right)=G^{*}\left(i\omega \right){\bar{\varepsilon }}_{0}e^{i\omega t}\;,$$

where $G^{*}\left(i\omega \right)$ is the complex dynamic modulus. Then, the constitutive relationship can be transformed into the frequency domain. The complex modulus is determined as

(4) $$G^{*}\left(i\omega \right)=G^{\prime }\left(\omega \right)+jG^{\prime \prime }\left(\omega \right)\;.$$

The components $G^{\prime }\left(\omega \right)$ and $G^{\prime \prime }\left(\omega \right)$ are called the storage and loss modulus, respectively, which are given as

(5) $$\begin{array}{l} G^{\prime }\left(\omega \right)=G_{0}-\sum_{i=1}^{N}\frac{G_{i}}{1+\tau_{i}^{2}\omega^{2}}\;,\\ G^{\prime \prime }\left(\omega \right)=\sum_{i=1}^{N}\frac{G_{i}\tau_{i}\omega }{1+\tau_{i}^{2}\omega^{2}}\;. \end{array}$$

The loss factor is defined as the ratio of the loss modulus over the storage modulus:

(6) $$\mu =\frac{G^{\prime \prime }\left(\omega \right)}{G^{\prime }\left(\omega \right)}\;.$$

### 2.2. Viscoelastic Material Properties of Additively Manufactured ABH

An Objet Connex 500 printer by Stratasys (Rehovot, Israel), which is capable of 3D printing abundant and various digital materials, was used in this study to fabricate the specimens. The printer works by accumulating small dots of resin that are then cured by ultraviolet light to create a homogeneous printed part. For this work, the digital materials were created using two base materials: VeroWhitePlus, which is a rigid opaque material, and TangoPlus, a rubber-like transparent material. Using these two base materials, the printer can produce ten different digital materials with varying stiffness [39]. In a previous work by the authors on 3D-printed functionally graded ABHs, specimens were fabricated using these ten digital materials [24]. Results showed that the ABHs with printed viscoelastic materials exhibited more effective wave energy dissipation compared to conventional ABHs due to the ABH feature and the viscoelastic material.

**Figure 1:**
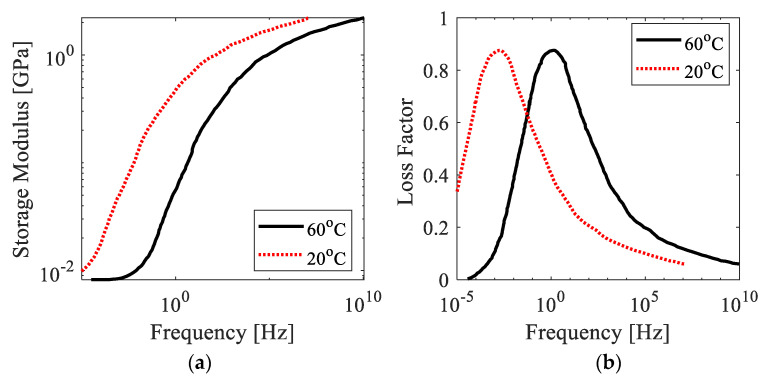
Material properties of VeroWhitePlus: (**a**) storage modulus, and (**b**) loss factor.

In order to determine the Prony series parameters, the complex modulus of the visco-elastic material was obtained by measuring the resulting strain under sinusoidal excitations using DMA. DMA measures the mechanical properties of materials as a function of temperature, frequency, and time. The base material VeroWhitePlus was used for fabricating the prototype of the ABH structure in this work. The material properties of the VeroWhitePlus at a reference temperature of 60 °C was tested and presented by Reichl [39]. It is important to note that changes in temperature can significantly alter both the storage modulus and the loss modulus of the material. The effects of both temperature and frequency can be combined into the reduced frequency parameter, denoted by $f_{r}\left(f,T\right)=f\cdot \alpha \left(T\right)$, where f and T represent the frequency and temperature at which the data was measured, respectively. The parameter is the shift factor, which satisfies an Arrhenius equation:

(7) $$\log\left[\alpha \left(T\right)\right]=T_{A}\left(\frac{1}{T}-\frac{1}{T_{0}}\right)\;,$$

where $T_{0}$ is an arbitrarily selected reference temperature, and $T_{A}$ is the Arrhenius temperature related to the activation energy. The Arrhenius temperature for the VeroWhitePlus is 16,183 K. Using Equation (7), the complex modulus of the material can be shifted to show its properties at any temperature. The storage modulus and loss factor of the material at room temperature (20 °C) were obtained and are shown in Figure 1. These data serve as the basis for obtaining the Prony coefficients of the viscoelastic material in Section 2.3, which follows, and are essential for obtaining accurate numerical results of the wave propagation characteristics in polymer ABH structures. It is evident that the material properties vary significantly with frequency.

### 2.3. Calibration of Prony Series

A proper model that accurately describes the viscoelastic behavior of the viscous material in ABH structures is essential for understanding the wave propagation and dissipation characteristics of such structures. Viscoelastic models based on the Prony series are commonly used due to their ease of implementation in finite element analysis. Abaqus also provides the option to input experimental data directly to the Prony series model, allowing for more accurate material representation. To obtain accurate results, Prony coefficients and relaxation times must be determined by fitting experimental data to either Equations (2) or (5). However, it should be noted that the accuracy of the model is highly dependent on the quality and accuracy of the experimental data. In addition, the choice of the number of terms in the Prony series can affect the accuracy of the model, with a larger number of terms typically resulting in better accuracy but at the cost of computational efficiency. Therefore, it is important to carefully select the number of terms in the Prony series to balance accuracy and efficiency in numerical simulations.

**Figure 2:**
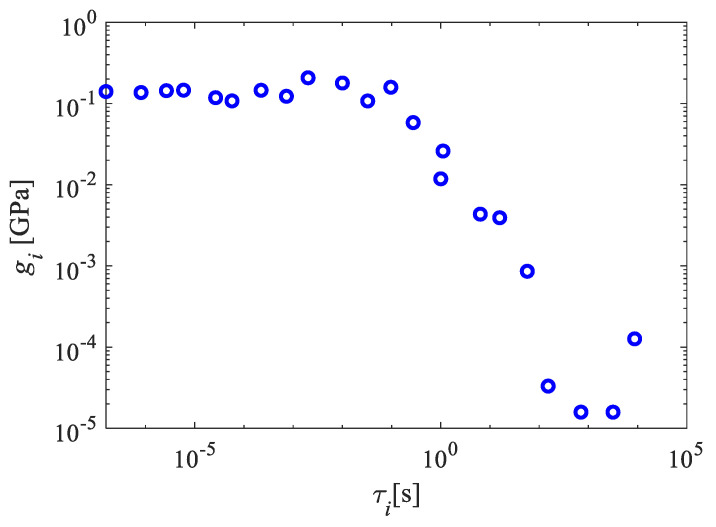
The parameters $G_{i}$ and $\tau_{i}$ of Prony series.

**Figure 3:**
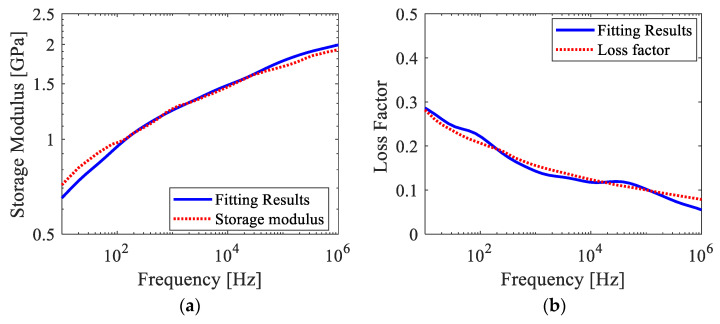
The fitting results and measured data of complex modulus for VeroWhitePlus at 20 °C: (**a**) storage modulus, and (**b**) loss factor.

The frequency ranges of interest for investigating dynamic characteristics of ABH structures is from 1 Hz to 1 MHz. In this work, the Prony coefficients and relaxation times were determined by fitting the experimental data of the complex modulus to the Prony series model within this frequency range. A two-step optimization method, combining particle swarm optimization and linear least square solver, was used to determine the coefficients. However, since the primary objective of this study was to investigate wave attenuation in viscoelastic ABH structures, the method for determining the coefficients of the Prony series is not discussed in detail here. Further details can be found in reference [38]. The fit was performed with equal weighting given to the real and imaginary parts, employing twenty-two exponential terms in Equation (5). The Prony series coefficients and relaxation times $G_{i}$ and $\tau_{i}$ for the Objet Connex material VeroWhitePlus at 20 °C are presented in Figure 2. The instantaneous modulus $G_{0}=2.07\;\mathrm{GPa}$, the storage modulus, and the loss modulus data with the curve fit using these Prony series parameters are shown in Figure 3. There was more than a three-fold difference in the modulus from 1 Hz to 1 MHz. The mean-square error (MSE) of the fitted storage modulus compared to the tested results was found to be 1.36%, indicating a small margin of error. It is important to note that the resulting parameters were inherent to the material, not the system. The quality of the Prony series obtained by fitting is further verified by experiments in Section 3.1.

## 3. Experimental Verification of Material Characterization

### 3.1. Experimental Set-Up and Finite Element Model

**Figure 4:**
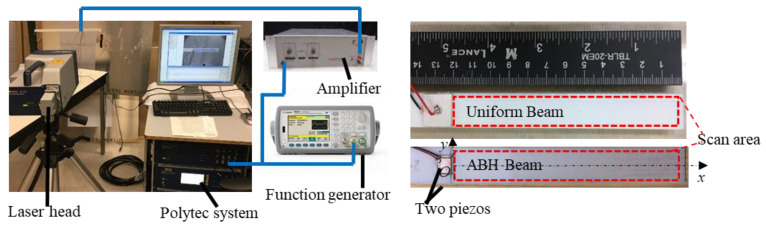
Experimental set-up and printed beam structures.

The Prony model’s credibility was verified in this section by comparing the simulation results with experimental measurements of wave propagation characteristics in a printed uniform beam using a SLDV system. The experimental system setup and examples of 3D-printed polymer beams are shown in Figure 4. The Polytec PSV-400 laser Doppler scanning system (Hörsching, Austria) was used to capture the waveform of the out-of-plane motion of the scanning area in specimens. Reflective paint was applied on the scanning area to improve the signal-to-noise ratio. An Agilent 33522A function generator was used to generate tone burst signals with various center frequencies. A Krohnhite 7500 amplifier (Brockton, MA, USA) was used to supply a power of gain of 10 to input to the piezo electric transducer (PZT).

In the experimental test, one end of the beam was clamped, and the other was free. The polymer beam with the ABH taper was also printed. The geometrically nonhomogeneous region of the ABH taper was described by the polynomial functions $h\left(x\right)={\left(x-0.12\right)}^{2}/3+0.0003,$ and (0.03 m≤x≤0.12 m). The width of the beams was 0.16 m. The total length of all the tested beams was 0.48 m. Two pieces of piezo ceramic plate were attached symmetrically on the beam 0.12 m away from the free end to excite the bending wave. The size of the piezo was 0.007 m×0.008 m×0.0002 m. The length of the scanning area was 0.12 m.

**Figure 5:**
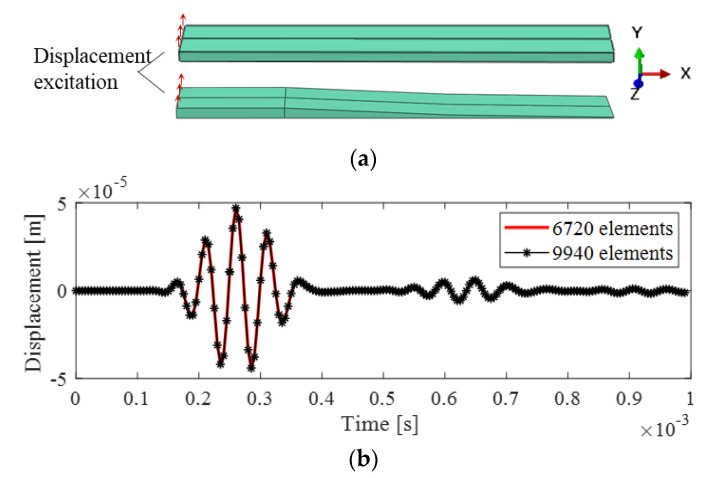
Models and responses in finite element simulations: (**a**) uniform beam and ABH beam; (**b**) displacement response.

The viscoelastic Prony series model and the parameters obtained in Section 2 above were utilized in this study to explore the wave propagation characteristics by the FE method. Numerical models were created and assembled using Abaqus. The material density was $1160\;\mathrm{kg}\cdot m^{-3}$, and the Poisson ratio was 0.35. Displacement excitations of a 5-cycle tone burst with various center frequencies were applied at the surface on the left side, as illustrated in Figure 5a, to generate plane incident waves. The three-dimensional finite element C3D20R was employed for simulations. The structural thickness at the tip of the ABH beam was extremely small, necessitating the use of higher-order elements to ensure computational accuracy. Additionally, to capture the wave phenomena effectively, the mesh size was carefully chosen, ensuring that the smallest wavelength included at least ten elements. In this study, the finite element (FE) models of the uniform beam and ABH beam consisted of 1600 and 6720 elements, respectively. To verify the mesh convergence, a study was conducted where an excitation signal with a center frequency of 20 kHz was applied to the ABH beam. The displacement response at *x* = 0.1 on the symmetry line of the bottom surface is plotted in Figure 5b. Comparing the result to those obtained using a refined mesh with 9940 elements, good consistency was observed.

### 3.2. Verification of Material Characterization

Wave propagation in the uniform beam was observed to experimentally verify the credibility of the parameters used in the Prony model. Firstly, the wave propagation in a uniform beam considering only the elastic component of the material with the instantaneous modulus $G_{0}$ was also simulated by the FE method as a reference.

**Figure 6:**
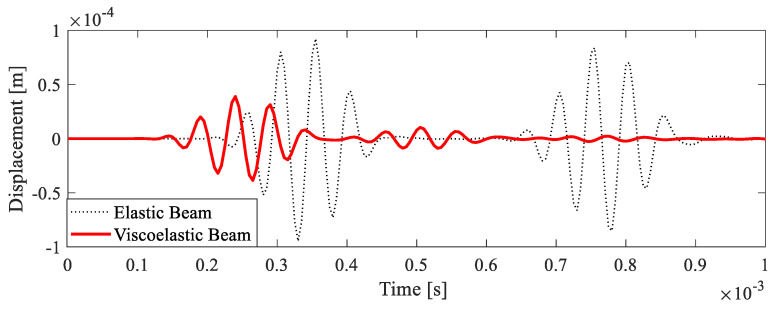
The displacement response of the uniform beam at a point 0.1 m from the free end on the symmetry line of the bottom surface.

The displacement response of the uniform beam at a point located 0.1 m from the free end on the symmetry line of the bottom surface was extracted, as shown in Figure 6. The center frequency of excitation was 20 kHz. The results demonstrated the attenuation of wave amplitude in the viscoelastic beam due to the dissipative mechanisms of the material. It was also observed that the wave velocity was affected by the viscous effect in the viscoelastic material as a result of the relaxation in the hold phase that was mentioned earlier. These results highlight the necessity of considering the viscoelastic effect when investigating the wave propagation and attenuation in polymer structures.

Experimental measurements and numerical simulations were compared to verify the accuracy of the Prony series model and the material parameters for estimating wave propagation characteristics in the printed beam structure. Excitation signals with two center frequencies (10 kHz and 20 kHz) were applied. Since, the wavelength depends on the frequency, geometry, and material character of the test specimen, it is convenient to observe a set of bending waves with these frequencies propagating along the length of the beam. However, considering the sensitivity of the test system, high quality tested data with a high signal-to-noise ratio can be obtained within the two center frequencies.

**Figure 7:**
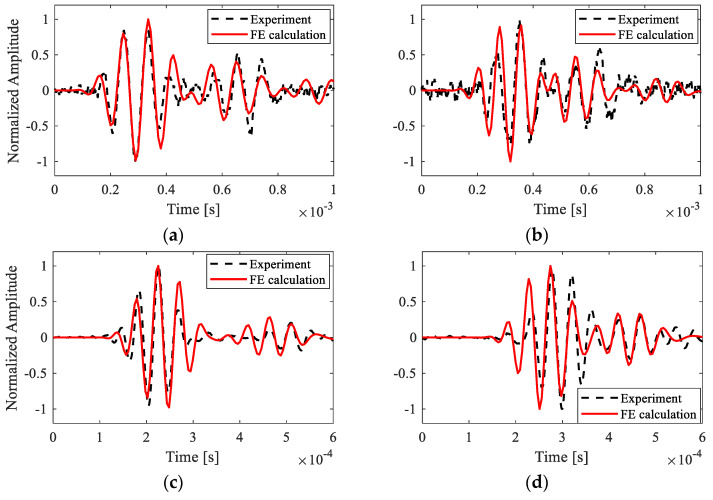
Responses at 2 points on the symmetry line of the bottom surface under excitations with different center frequencies: (**a**) point A with 10 kHz; (**b**) point B with 10 kHz; (**c**) point A with 20 kHz; (**d**) point B with 20 kHz.

**Figure 8:**
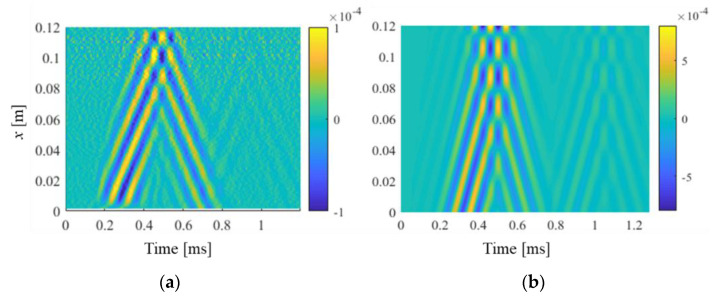
Dynamic responses of the symmetric line on the bottom surface in the space–time domain: (**a**) measured signals; (**b**) FE simulation.

Signals of the dynamic response at two random points on the symmetry line of the beam bottom surface were extracted and normalized to their maximum value. The two points were marked as point A (*x* = 0.11 m away from the free end) and point B (*x* = 0.075 m from the free end). As shown in Figure 7, the time–domain response demonstrated that wave amplitudes were attenuated after reflecting from the free end, with higher frequency waves experiencing more pronounced attenuation. Comparing the result in Figure 7c,d to that in Figure 7a,b, the arrival time of waves was also delayed with a higher frequency, indicating a smaller wave velocity due to the viscoelastic effect. Furthermore, the out-of-plane response to the transient excitation with a center frequency of 10 kHz was extracted over the line of symmetry on the bottom surface to analyze wave propagation in the printed beam, as shown in Figure 8. The results demonstrate reasonable consistency between simulations and experimental data, indicating the validity and applicability of the Prony series model and material parameters for estimating wave propagation characteristics in the printed beam structure.

## 4. Results and Discussion

Using the calibrated viscoelastic model in FE simulations and experimental measurements with the SLDV system, we investigated wave attenuation in the printed polymer ABH structure while considering the viscoelastic effects. Additionally, we discussed the effect of loading frequency on the wave attenuation.

### 4.1. Wave Reflection Coefficient in Printed Specimen

**Figure 9:**
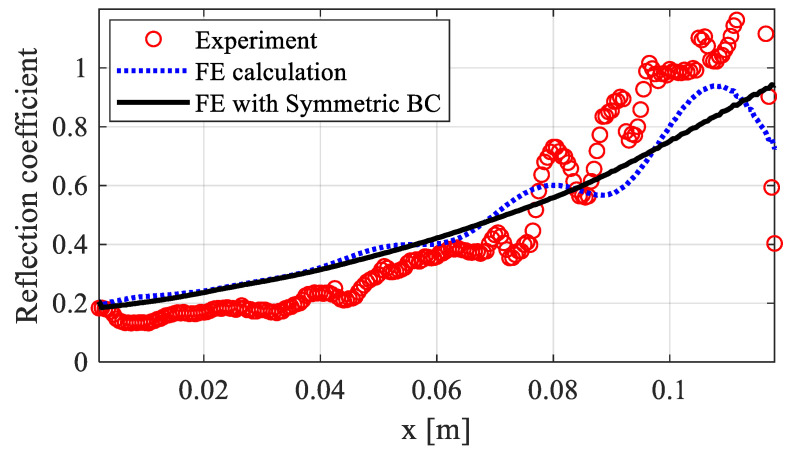
Reflection coefficients along the length of the printed beam.

To evaluate the wave attenuation in printed beam structures and investigate the effect of loading frequency, the incident and reflected waves were separated to obtain the spatial distribution of the reflection coefficient [4]. This coefficient was calculated as the ratio of the amplitude of the reflected wave to that of the incident wave. Figure 9 shows the reflection coefficient along the length of the printed beam based on the dynamic responses in the space–time domain obtained from simulations and experimental measurements. The experimental results showed some fluctuation due to the boundary effect, which was previously reported in the authors’ previous work [24]. The application of a symmetric boundary condition at two surfaces perpendicular to the *z*-axis in the FE simulation eliminated this effect and yielded a perfect beam model, as shown in Figure 9. The reflection coefficient gradually increased from 0.2 at *x* = 0 m to 1 at the free tip, demonstrating the attenuation process of the wave along the direction of wave propagation in the printed beam. The FE calculations showed good consistency with the experimental result. For convenience, the symmetric boundary condition was applied in the following numerical simulations.

### 4.2. Wave Attenuation and Influence of Load Frequency in Printed ABH Beam

**Figure 10:**
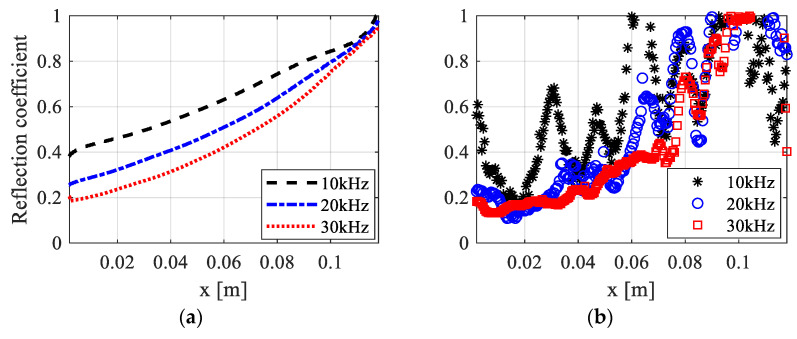
Reflection coefficients in the uniform beam with three different center frequency of incident waves: (**a**) FE numerical results; (**b**) experimental results.

**Figure 11:**
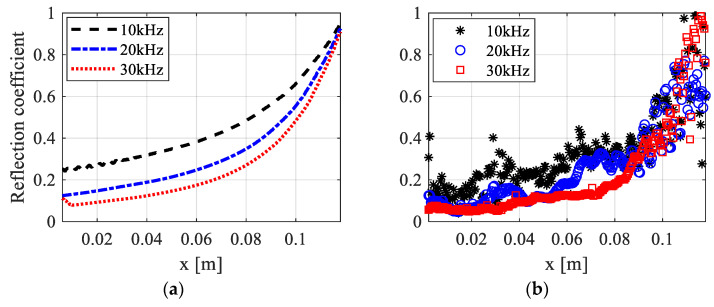
Reflection coefficients in the ABH beam with three different center frequencies of incident waves: (**a**) FE numerical results; (**b**) experimental results.

Based on the model and analysis discussed in the previous section, this section discusses the wave attenuation effect and influence of load frequency in the printed ABH beam. Reflection coefficients in the ABH beam and the uniform beam with three different center frequencies of incident waves are presented in Figure 10 and Figure 11. The reflection coefficients in both specimens, as revealed by FE simulations and experiments, decrease with increased frequency. It is worth noting that the loss factor in the VeroWhitePlus material decreases with increased frequency, as illustrated in Figure 3. However, the viscoelastic mechanism of the material still makes energy dissipation more efficient for waves with higher frequencies. Although fluctuations in reflection coefficients exist in experimental beam structures due to the boundary effect, there is a fairly good relevance to numerical results.

Comparing the reflection coefficient in the ABH beam to that in the uniform beam, a more evident wave attenuation is obtained for the same load frequency. The gradient of the curve of the reflection coefficient indicates the degree of energy dissipation at the current position after the primary reflection from the tip. It illustrates that a more efficient wave energy dissipation is achieved by a smaller amount of viscoelastic material with ABH features. The reflection coefficient decreases to almost half in the ABH beam compared to that in the uniform beam. In short, the effective wave attenuation effect in the 3D-printed ABH beam is obtained. The wave propagation characteristics can be accurately predicted by the numerical simulation using the Prony series model, considering the viscoelastic property of the material. Results demonstrate the potential of viscous ABH structures for vibration suppression as lightweight damping.

## 5. Conclusions

In this study, the objective is to investigate the wave attenuation effect in additively manufactured polymer ABH structures considering viscoelastic effects. The Prony series model is employed to accurately describe the viscoelastic behavior of the material, incorporating frequency-dependent changes in the damping properties. Experimental dynamic mechanical analysis is conducted to determine the parameters of the Prony model, which are then applied to finite element models for simulating the wave attenuation characteristics in polymer ABH structures. The numerical results are validated through experimental measurements using a scanning laser doppler vibrometer system.

The findings of this study demonstrate the effectiveness of the Prony series model in accurately predicting the wave attenuation behavior in polymer ABH structures. The simulations show good consistency with the experimental results, confirming the validity of the model. The study also highlights the importance of considering the viscoelastic effects of the material in investigating wave energy dissipation. The ABH effect in the printed polymer structure, particularly with regard to the wave attenuation, is influenced by both the viscoelastic material properties of the polymer and the geometric features of the ABH. Due to the relaxation of the viscoelastic material properties in the printed polymer, the attenuation of wave amplitude and the variation in wave velocity result as well. The reflection coefficients showed a more efficient wave energy dissipation, especially near the tip of the wedge, due to the geometric features of the ABH. The wave attenuation is effectively enhanced in the printed ABH structure with a lesser amount of polymer material. The influence of the load frequency on wave attenuation in the printed ABH beam is investigated, and the results show that lower reflection coefficients are achieved in both the ABH beam and the uniform beam with higher frequencies due to the viscoelastic mechanism of the material, which is validated in both numerical simulations and experimental results. This behavior is attributed to both the viscoelastic properties of the material and the geometric features of the ABH, such as the tapering edge or locally thin areas.

Despite the successful investigation of wave attenuation in polymer ABH structures, there are a few limitations to be acknowledged. The specific material used in this study might have limitations in terms of its performance in wave attenuation. Therefore, further research should explore different materials to optimize the ABH effect.

## Data Availability

Not applicable.
